# Position specific physical demands in different phases of competitive matches in national level women’s football

**DOI:** 10.5114/biolsport.2023.118337

**Published:** 2022-09-15

**Authors:** Juho K Mäkiniemi, Eero HJ Savolainen, Taija Finni, Johanna K Ihalainen

**Affiliations:** 1 Faculty of Sport and Health Sciences, University of Jyväskylä, Finland

**Keywords:** Women’s football, Physical performance, Physical demands, Match analysis, Position-specific, National level

## Abstract

The purpose of the present study was twofold: to investigate position-specific physical match demands of national level women’s football; and to examine if demands change during a match (comparison between first and second half and in 15-minute intervals). Seven teams from the Finnish National League participated in the study. Eighty-five players met the inclusion criteria, and a total of 340 individual match observations from 68 individual matches were included for analysis. The Polar Team Pro -player tracking system (with 10 Hz GPS units, including 200 Hz tri-axial accelerometer, gyroscope, magnetometer and HR monitor) was used to assess positional data and HR response of the players. This study demonstrated that women’s national level football matches place a range of physical demands on players, which in general were highest for wide midfielders, and lowest for central defenders. Wide midfielders and forwards performed significantly more ‘very high-speed’ running, sprinting, accelerations, and decelerations than other outfield positions (p < 0.05). HRmean varied from 84–87% of HRmax and was significantly lower for central defenders than central midfielders (p < 0.001). External load variables varied during a match and generally decreased especially after 60 minutes of play compared to first 15-min period of the match. Present study showed that national level women football players’ positional differences in match demands are similar to those reported with elite players in previous studies. On national level, players’ physical performance tended to decrease towards the end of the match, especially in terms of total distance (~10%), high-speed running (~20%), and decelerations (~20%).

## INTRODUCTION

A comprehensive understanding of position and match-specific physical demands is vital to apply a systematic approach to training that reflects the physical load players will face during games [1, 2]. Studies have shown that physical demands are highest in the beginning of the match and decrease towards the end of the match [3–6]. To date there are only a limited number of studies that have investigated position-specific physical demands during different phases of a match. Furthermore, scientific literature has focused primarily on physical demands of elite players, leaving a gap in the literature that is essential for understanding and enhancing player development [2].

Previous studies have shown that elite female players cover a total distance (TD) of 9–11 kilometres [1, 4, 7–11]. The highest TD recorded has been observed in central midfielders (CM) and the lowest in central defenders (CD) [1, 11, 12]. Dividing a match into 15-minute periods allows for the identification of changes in movement patterns and helps to understand the impact of fatigue on performance [13]. In a sample of elite level outfield players, a significant decrease in TD has been observed at 60–75 minutes and 75–90 minutes compared to 0–15 minutes [4]. Position-specific analyses have shown that TD decreases from the first to last 15 minutes by 5–13% in all outfield positions except forwards (FW) [14].

Instead of TD, volume of high-intensity running appears to better reflect the requirements of the game [7, 15–17] as elite athletes tend to cover larger distances at high intensities than domestic athletes [2]. Regrettably, there is inconsistency in women’s football research concerning speed zones and their definitions [1]. High-speed running (16–20 km/h) and sprinting (> 20 km/h) zones have been used in the previous literature [8, 12, 18–20]. Recent studies [10, 11] however, have used thresholds including 12.5 km/h for high-speed running (HSR), 19.0 km/h for very high-speed running (VHSR), and 22.5 km/h for sprinting (SPR) as suggested by Park et al. [21]. A match analysis of the FIFA Women’s World Cup 2019 used almost identical thresholds for the highest three zones: 13 km/h (Zone 3), 19 km/h (Zone 4), and 23 km/h (Zone 5) [1]. Elite level female players have been shown to complete from 1676–2749 m of HSR, 349–666 m of VHSR and 82–255 m of SPR depending on the playing position [1, 11].

The volume of high-intensity running has been shown to vary considerably between different positions [1, 8, 9, 12, 22]. CD cover the lowest distances in high-intensity zones [1, 11, 12, 22] whereas CM run substantially more HSR than other players [1, 11, 12], and wide midfielders (WM) perform the highest volume of VHSR and SPR. Full backs (FB) and FW also cover a higher volume of VHSR and SPR than CD and CM [1, 11, 12].

A decrease of up to a range of 25–35% in HSR has been observed between the first and last 15 minutes of the match in a sample of all positions [3, 4, 6, 9]. Panduro et al. [14] showed that HSR and VHSR of all outfield positions decreased significantly in the last 15-minute period of a match compared to the first 15-minute period. Similar results were found with SPR in all positions except FW [14]. Evidence from other studies, however, has shown that SPR distance does not change significantly between the 15-min periods, unlike between the first and second halves [4, 5].

Acceleration and deceleration parameters in women’s football have received only limited attention, despite metabolic consumption being higher compared to steady running [23, 24]. Indeed, a number of accelerations and decelerations vary considerably between studies [7, 8, 25, 26] and a common definition for acceleration and deceleration thresholds does not exist. Recent studies in men have used thresholds of > 2 m/s^2^ and < -2 m/s^2^ with a range of 76–94 accelerations and 54–99 decelerations per match [23, 27]. In women’s international level matches, ~160 accelerations (> 2.26 m/s^2^) have been reported [8, 25]. International level players tend to perform more accelerations and decelerations during match [7] than national level players [28] and no major position-specific differences have been observed [7, 26]. A recent study [14] reported a higher number of moderate accelerations (1.50–2.99 m/s^2^) and decelerations (-1.50 – -2.99 m/s^2^) for CM and WM than CD. They also reported that the total number of accelerations and decelerations was significantly lower during the match’s last 15-minutes period compared to the first 15-min in all outfield positions [14], but their comparison was limited only to the first and the last 15-min periods of a match.

Heart rate (HR) measurement is often used as an indirect measure of intensity during training or matches and is used to observe the internal load of players [29]. As a result of short and intense efforts, the average HR of women footballers during matches has been reported to be 84–89% and the HR peak 98–100% [3, 30, 31]. A HR of about 85% of maximum HR corresponds to about 75% of maximal oxygen uptake [15, 17, 27, 32]. This indicates that players experience a high aerobic load throughout the match [33]. Average HR were reported significantly higher in the second half in all outfield positions except FW, however there were no differences in average HR between first and last 15 minutes period in the match in any position [14].

Scientific literature is still lacking research of women’s national level position-specific match demands. Therefore, purpose of the study is to (1) identify positional differences in match demands of national level female football players and to (2) investigate if positional match demands change during a match (between 1^st^ and 2^nd^ halves or between 15-minute periods).

## MATERIALS AND METHODS

During the 2020 season, 10 teams from the highest Finnish women’s football league were recruited and 7 teams participated to in this observational study. A minimum of four and maximum of nine matches for each team were included for analysis with an average of 7.6 ± 1.7 matches per team. Only data from players completing full matches (90 min) were included. Of the eligible 125 players in the league who provided consent for participation, a total of 85 players (age: 23.7 ± 4.1 years, height: 168.0 ± 6.2 cm, weight: 64.3 ± 8.1 kg) were accepted in the analysis. On average 4.0 ± 2.6 matches per player were analysed (range 1–9 matches). In total, 340 match observations were completed from 68 matches. All participants provided written informed consent prior to participating the study. The study was conducted according to the declaration of Helsinki and was approved by the ethics committee of the University of Jyväskylä (1064/13.00.04.00/2020).

Positional data and HR response were collected using Polar Team Pro units (Polar Electro Oy, Kempele, Finland) with GPS (Global Positioning System) sampling at a 10 Hz frequency, 200 Hz tri-axial accelerometer, gyroscope, magnetometer and HR monitor, and analysed using proprietary software. According to Scott et al. [34], all GPS devices can determine distances accurately during team sport simulations (trainings and matches) and can therefore reasonably be used in team sports. Good to moderate reliability (< 5% CV) and validity for total distance, linear running and team sport simulation circuit have been shown for the Polar Team Pro system [35].

Players were divided into five distinct outfield playing positions for analysis: central defender (CD, number of observations 86), fullback (FB, 40), central midfielder (CM, 98), wide midfielder (WM, 31) and forward (FW, 48). Full-game data is presented to describe the complete match demands of the game for each position. Moreover, to examine possible changes in demands during matches, data from first to second half and between 15-minute periods were compared and analyzed [5, 7]. In addition, full-game values and values for each half are presented for goalkeepers.

Prior to starting analysis, player data were excluded in cases where it was missing fully or partly from the match, i.e., in situations where the player tracking system did not record data for the whole match (n = 8). During data review, error peaks were removed from peak heart rates (e.g., player heart rate peak well above 100% of HRmax) and peak speeds (e.g., all speed peaks above 35 km/h).

Mean and peak HR were analysed based on a continuous measurement throughout the match, excluding the half-time break. HRmax was defined as each individual player’s maximum HR collected during the measurement period. Mean and peak HRs are presented as a percentage relative to maximum heart rate (% HRmax). Time spent in different HR zones (as a percentage of total playing time) was classified as follows: HRZ1 (50–59% of maximum heart rate), HRZ2 (60–69%), HRZ3 (70–79%), HRZ4 (80 –89%), HRZ5 (90–100%). Time spent in HRZ1 and HRZ2 was limited and thus is not presented in the results.

Positional analysis of distances covered in different speed zones, peak speed, and total distances were extracted using proprietary software. The movement velocity of the players was divided into five speed zones: speed zone 1 (< 7 km/h), speed zone 2 (7–13 km/h), speed zone 3 (HSR, 13–19 km/h), speed zone 4 (VHSR, 19–23 km/h), and speed zone 5 (SPR, > 23 km/h) [1]. The distances covered in HSR, VHSR and SPR are presented. Accelerations and decelerations were summarized when the acceleration or deceleration speed exceeded the threshold of ± 2.00 m/s^2^ as defined in previous research [5, 24].

Data analyses were performed using SPSS software (version 26, IBM SPSS Statistics, Chicago, IL, USA). The threshold for statistical significance was set at p < 0.05. Data are presented as mean ± standard deviation unless otherwise stated. The Shapiro-Wilk test was used to test for normal distribution. If the requirement for a normal distribution was not met, variables were removed from analysis (e.g., 15 min period HRZ1 & HRZ2) or the variables were combined (e.g., in 15-minute periods the VHSR and SPR were combined) to meet the assumption of a normal distribution.

For each full match, position-specific differences were assessed using one-way ANOVA. Goalkeepers were excluded from the statistical analysis. To determine whether there was a position-specific significant interaction between first and second half, as well as between 15-minute periods, a two-way ANOVA (position*time) was used with repeated measures. If a significant interaction was observed, a one-way ANOVA was used between the first and second half or 15-minute period values. Effects were further analysed by performing Bonferroni corrected pairwise comparisons.

## RESULTS

Table 1 shows positional differences in full match demands. WM and CM had highest TD covered, while CD covered the significantly lower TD compared to other outfield positions. WM and FW players had significantly more VHSR, SPR, number of accelerations and decelerations than other outfield positions, while CD results in same parameters were significantly lower than other positions.

**TABLE 1:** Positional differences in match demands.

	GK	CB	FB	CM	WM	FW
TD [m]	4788±389	9275 ± 566 ^b,c,d,e^	10063 ± 762 ^a,c,d^	10634 ± 707 ^a,b^	10797 ± 681 ^a,b,e^	10295 ± 731 ^a,d^
HSR [m] (13-19 km/h)	148 ± 84	1515 ± 289 ^b,c,d,e^	1785 ± 336 ^a,c,d,e^	2251 ± 470 ^a,b,e^	2233 ± 449 ^a,b^	2027 ± 365 ^a,b,c^
VHSR[m] (19-23 km/h)	10 ± 15	215 ± 71 ^b,c,d,e^	317 ± 100 ^a,d,e^	326 ± 156 ^a,d,e^	445 ± 113 ^a,b,c^	405 ± 126 ^a,b,c^
SPR [m] (> 23 km/h)	2 ± 8	45 ± 31 ^b,d,e^	82 ± 52 ^a,d,e^	61 ± 62 ^d,e^	136 ± 76 ^a,b,c^	115 ± 70 ^a,b,c^
Peak speed [km/h]	20.1 ± 2.7	26.3 ± 1.7 ^b,d,e^	27.4 ± 1.9 ^a,c^	25.8 ± 2.0 ^b,d,e^	27.7 ± 1.8 ^a,c^	27.5 ± 1.9 ^a,c^
Accelerations [n] (> 2.00 m/s^2^)	25.8 ± 9.2	67.5 ± 16.3 ^c,d,e^	74.3 ± 16.8 ^d,e^	82.5 ± 20.9 ^a,d,e^	98.3 ± 19.1 ^a,b,c^	92.9 ± 21.6 ^a,b,c^
Decelerations [n] (< -2.00 m/s^2^)	24.9 ± 11.2	79.0 ± 16.5 ^c,d,e^	90.3 ± 20.6 ^c,d,e^	105.1 ± 23.4 ^a,b,c,d,e^	121.5 ± 18.3 ^a,b,c^	118.4 ± 26.3 ^a,b,c^
HR mean (%/ HR_max_)	75.2 ± 5.2	84.2 ± 5.3 ^c^	86.0 ± 3.9	86.9 ± 3.5 ^a^	85.2 ± 4.4	85.8 ± 3.3
HR peak (%/ HR_max_)	90.6 ± 4.6	96.2 ± 2.6	97.5 ± 2.8	97.1 ± 2.3	97.5 ± 2.4	97.3 ± 2.8
HRZ3 (70-80%/ HR_max_)%/ total time	41.4 ± 13.5	17.0 ± 11.6 ^c^	13.4 ± 9.2	10.9 ± 8.3 ^a^	14.1 ± 8.8	12.8 ± 8.1
HRZ4 (80-90%/ HR_max_)%/ total time	26.4 ± 16.8	48.0 ± 13.3	49.3 ± 14.7	43.2 ± 14.2 ^e^	48.3 ± 15.1	54.7 ± 15.6 ^c^
HRZ5 (90-100%/ HR_max_)%/ total time	3.7 ± 22.5	27.9 ± 20.9 ^c^	33.5 ± 20.3	42.1 ± 21.5 ^a,e^	31.7 ± 21.8	28.6 ± 21.3 ^c^

TD = total distance covered during match, HSR = High-speed running distance covered during match, VHSR = very high-speed running distance covered during match, SPR = sprint distance covered during match, HR = heart rate, HRZ3–5 = heart rate zones 3–5. GK = goalkeepers, CD = central defenders, FB = full backs, CM = central midfielders, WM = wide midfielders, FW = forwards. Statistically significant difference (p < 0.05) compared to

a = central defenders,

b = full backs,

c = central midfielders,

d = wide midfielders,

e = forwards.

Mean HR was 85.7% of HRmax. The only statistically significant difference observed was a higher average HR in CM compared to CD. Players spent about 50% of the match time on HRZ4. CM spent significantly more time in HRZ5 compared to CD and FW. Only 3–6% of total match time was spent in HRZ1 and HRZ2.

Table 2 shows match demands during the first and second halves. Statistically significant decrement in external load was observed only in TD and HSR of CM, while VHSR distance of CD increased significantly from first to second half. In terms of internal load, time spent in HRZ5 decreased significantly in FB and CM. Time spent in HRZ3 increased significantly in FB, CM and WM.

**TABLE 2:** Positional match demands during first and second half

	Half	GK	CD	FB	CM	WM	FW
TD [m]	1^st^	2416 ± 200	4642 ± 390	5075 ± 385	5373 ± 390*	5396 ± 307	5205 ± 371
	2^nd^	2372 ± 250	4632 ± 380	4988 ± 449	5261 ± 363	5401 ± 417	5090 ± 401
HSR [m] (13–19 km/h)	1^st^	65 ± 37	759 ± 160	901 ± 168	1169 ± 264*	1122 ± 246	1042 ± 179
	2^nd^	83 ± 56	756 ± 158	885 ± 195	1083 ± 235	1111 ± 240	985 ± 200
VHSR[m] (19–23 km/h)	1^st^	2 ± 4	100 ± 38*	165 ± 59	165 ± 80	218 ± 69	203 ± 78
	2^nd^	8 ± 13	115 ± 49	153 ± 63	162 ± 87	226 ± 72	202 ± 72
SPR [m] (> 23 km/h)	1^st^	0 ± 1	23 ± 20	41 ± 29	30 ± 35	65 ± 41	57 ± 45
	2^nd^	2 ± 7	22 ± 19	41 ± 27	31 ± 34	71 ± 44	58 ± 40
Peak speed [km/h]	1^st^	18.1 ± 2.1	25.3 ± 1.9	26.4 ± 2.0	25.1 ± 2.2	26.9 ± 1.8	26.4 ± 2.1
	2^nd^	19.8 ± 3.0	25.3 ± 1.9	26.5 ± 2.3	25.0 ± 2.0	26.8 ± 2.0	26.7 ± 2.0
Accelerations [n] (> 2.00 m/s^2^)	1^st^	12.5 ± 4.8	33.4 ± 11.4	37.7 ± 8.7	41.7 ± 11.4	49.1 ± 11.9	47.4 ± 11.8
	2^nd^	13.3 ± 6.2	34.0 ± 11.6	36.6 ± 11.2	40.9 ± 11.6	49.2 ± 10.4	45.5 ± 12.8
Decelerations [n] (< -2.00 m/s^2^)	1^st^	12.5 ± 4.8	39.5 ± 9.0	46.2 ± 10.8	53.8 ± 12.5	62.8 ± 11.0	60.9 ± 13.3
	2^nd^	13.3 ± 6.2	39.5 ± 10.0	44.1 ± 11.4	51.3 ± 12.9	58.8 ± 10.2	57.5 ± 15.1
HR mean [%HR_max_]	1^st^	75.4 ± 5.4	84.4 ± 5.6	86.9 ± 3.9	87.9 ± 3.3*	86.1 ± 4.5	86.2 ± 3.8
	2^nd^	75.1 ± 5.4	83.8 ± 5.8	85.2 ± 4.4	85.9 ± 4.4	84.1 ± 4.6	85.1 ± 3.5
HR peak [%HR_max_]	1^st^	89.5 ± 4.6	95.4 ± 2.9	96.6 ± 2.7	96.5 ± 2.3	97.1 ± 2.2	96.2 ± 3.0
	2^nd^	89.4 ± 5.4	95.2 ± 2.7	96.0 ± 3.2	96.1 ± 2.6	96.5 ± 2.6	96.2 ± 3.5
HRZ3 (70–80%/HR_max_) %/ total time	1^st^	41.6 ± 13.8	16.1 ± 12.3	10.0 ± 8.9*	8.4 ± 8.1*	11.0 ± 8.9*	11.2 ± 8.9
	2^nd^	41.4 ± 15.1	18.0 ± 12.6	16.7 ± 10.7	13.4 ± 10.0	17.6 ± 11.5	14.4 ± 9.3
HRZ4 (80–90%/HR_max_) %/ total time	1^st^	28.1 ± 20.8	47.8 ± 15.4	47.1 ± 16.4	41.3 ± 16.7	45.9 ± 18.3	53.7 ± 17.3
	2^nd^	24.8 ± 19.2	48.3 ± 14.3	51.5 ± 14.9	45.1 ± 14.1	51.2 ± 14.9	56.1 ± 16.7
HRZ5 (90–100%/HR_max_) %/ total time	1^st^	3.0 ± 6.1	29.7 ± 23.0	39.3 ± 22.5*	47.7 ± 22.9*	37.5 ± 24.3	31.6 ± 23.7
	2^nd^	4.2 ± 9.0	26.4 ± 20.5	28.1 ± 21.1	36.8 ± 22.3	26.2 ± 21.0	26.0 ± 20.6

TD = total distance covered during match, HSR = High-speed running distance covered during match, VHSR = very high-speed running distance covered during match, SPR = sprint distance covered during match, HR = heart rate, HRZ3–5 = heart rate zones 3–5. GK = goalkeepers, CD = central defenders, FB = full backs, CM = central midfielders, WM = wide midfielders, FW = forwards.

* = Statistically significant difference (p < 0.05) compared to second half.

Table 3 shows match demands in 15-minute periods. In TD each position decrement from 0–15 minutes to 60–75 minutes and to 75–90 minutes periods was statistically significant. In HSR, the decrement towards the end of the match was highest and statistically significant compared to the 0–15 minutes in center positions (CD, CM and FW, p < 0.05). In combined VHSR & SPR distance only WM had statistically significant differences between 15-min periods. The number of decelerations decreased significantly towards the end of the match in all positions, but the number of accelerations only decreased significantly in only CM.

**TABLE 3:** Positional match demands in 15 minutes intervals

	Period	CD	FB	CM	WM	FW
Total distance [m]	*0–15 min*	1544 ± 118 ^2,3,4,5,6^	1654 ± 131 ^5,6^	1789 ± 143 ^2,3,4,5,6^	1782 ± 140 ^5,6^	1730 ± 160 ^2,5,6^
	*15–30 min*	1449 ± 122 ^1,6^	1602 ± 134 ^5^	1684 ± 142 ^1,5,6^	1676 ± 144	1619 ± 129 ^1,6^
	*30–45 min*	1458 ± 138^1,5,6^	1599 ± 159 ^5^	1692 ± 150 ^1,5,6^	1694 ± 164	1646 ± 142 ^5,6^
	*45–60 min*	1479 ± 129 ^1,5,6^	1576 ± 143	1676 ± 133 ^1,5,6^	1714 ± 147	1645 ± 130 ^5,6^
	*60–75 min*	1396 ± 129 ^1,3,4^	1500 ± 159 ^1,2,3^	1596 ± 150 ^1,2,3,4^	1654 ± 161 ^1^	1543 ± 160 ^1,3,4^
	*75–90 min*	1383 ± 125 ^1,2,3,4^	1516 ± 168 ^1^	1570 ± 138 ^1,2,3,4^	1600 ± 147 ^1^	1518 ± 145 ^1,2,3,4^

HSR [m] (13–19 km/h)	*0–15 min*	282 ± 71^2,3,4,5,6^	303 ± 61	422 ± 106 ^2,3,4,5,6^	400 ± 105 ^6^	378 ± 86 ^2,3,5,6^
	*15–30 min*	233 ± 65 ^1^	292 ± 76	367 ± 91 ^1,5,6^	347 ± 96	315 ± 63 ^1^
	*30–45 min*	224 ± 69 ^1^	277 ± 76	346 ± 96 ^1^	338 ± 97	314 ± 72 ^1^
	*45–60 min*	248 ± 70 ^1^	294 ± 79	363 ± 92 ^1,6^	356 ± 89	337 ± 73 ^6^
	*60–75 min*	223 ± 61 ^1^	265 ± 72	327 ± 83 ^1,2^	335 ± 90	294 ± 81 ^1^
	*75–90 min*	226 ± 58 ^1^	258 ± 75	310 ± 84 ^1,2,4^	326 ± 91 ^1^	284 ± 82 ^1,4^

VHSR & SPR [m] (≥ 19 km/h)	*0–15 min*	44 ± 28	69 ± 37	72 ± 43	119 ± 46 ^2,3,4^	97 ± 46
	*15–30 min*	38 ± 26	72 ± 30	62 ± 46	82 ± 38 ^1^	74 ± 50
	*30–45 min*	39 ± 26	61 ± 31	55 ± 45	72 ± 37 ^1^	79 ± 41
	*45–60 min*	44 ± 29	60 ± 32	59 ± 43	88 ± 39 ^1^	87 ± 46
	*60–75 min*	41 ± 25	60 ± 36	60 ± 40	95 ± 34	79 ± 31
	*75–90 min*	42 ± 28	56 ± 36	59 ± 44	94 ± 42	75 ± 42

Peak speed [km/h]	*0–15 min*	23.5 ± 2.1	24.3 ± 2.6	23.8 ± 2.3	25.7 ± 2.3	25.2 ± 2.1
	*15–30 min*	23.5 ± 2.6	24.7 ± 2.2	23.5 ± 2.4	25.0 ± 2.3	24.2 ± 2.7
	*30–45 min*	23.4 ± 2.5	24.6 ± 2.0	23.2 ± 2.4	25.2 ± 2.3	24.7 ± 2.0
	*45–60 min*	23.1 ± 2.2	23.9 ± 2.1	23.4 ± 2.2	25.1 ± 2.0	25.1 ± 2.5
	*60–75 min*	23.6 ± 2.2	24.4 ± 2.6	23.4 ± 2.2	25.8 ± 1.9	25.2 ± 2.0
	*75–90 min*	23.6 ± 2.3	24.4 ± 3.0	23.4 ± 2.4	25.0 ± 2.3	24.7 ± 2.2

Accelerations [n] (> 2.00 m/s^2^)	*0–15 min*	12.0 ± 3.9 ^3^	13.0 ± 4.2	15.1 ± 4.9 ^2,3,5,6^	16.7 ± 5.5	16.8 ± 4.7
	*15–30 min*	10.6 ± 4.1	11.9 ± 3.8	12.7 ± 4.5 ^1^	14.8 ± 4.9	14.2 ± 4.9
	*30–45 min*	9.9 ± 3.2 ^1^	11.2 ± 4.0	12.6 ± 4.8 ^1^	15.6 ± 5.0	14.9 ± 4.4
	*45–60 min*	10.6 ± 4.3	11.0 ± 3.9	13.6 ± 4.7	16.0 ± 4.7	14.9 ± 4.7
	*60–75 min*	10.2 ± 4.1	11.6 ± 4.1	11.8 ± 4.5 ^1^	14.4 ± 3.6	13.9 ± 5.1
	*75–90 min*	10.5 ± 4.6	11.1 ± 4.3	12.2 ± 5.4 ^1^	15.0 ± 5.3	13.9 ± 4.5

Decelerations [n] (< -2.00 m/s^2^)	*0–15 min*	13.6 ± 4.1 ^6^	16.2 ± 5.2 ^5,6^	18.6 ± 5.4 ^5,6^	21.5 ± 5.4 ^5,6^	21.2 ± 4.7 ^2,5,6^
	*15–30 min*	12.5 ± 3.8	14.2 ± 4.5	16.8 ± 5.1	18.8 ± 4.5	17.5 ± 4.9 ^1^
	*30–45 min*	12.2 ± 3.8	14.1 ± 4.3	16.6 ± 4.8	20.3 ± 5.8	19.8 ± 4.4 ^5^
	*45–60 min*	13.1 ± 4.4	14.9 ± 4.2	17.6 ± 5.6 ^5,6^	18.8 ± 5.6	19.6 ± 4.7 ^5^
	*60–75 min*	12.0 ± 4.0	12.9 ± 4.1 ^1^	14.8 ± 4.5 ^1,4^	17.5 ± 3.6 ^1^	16.3 ± 5.1 ^1,3,4^
	*75–90 min*	11.6 ± 3.7 ^1^	13.0 ± 4.3 ^1^	15.1 ± 4.9 ^1,4^	17.7 ± 4.7 ^1^	17.8 ± 4.5 ^1^

HR mean [%HR_max_]	*0–15 min*	84.3 ± 4.9	86.3 ± 3.9	86.9 ± 3.8 ^3^	85.8 ± 4.5	85.4 ± 3.8
	*15–30 min*	84.8 ± 6.0	87.5 ± 4.4 ^4^	88.0 ± 3.7 ^4,6^	86.3 ± 5.1	86.3 ± 3.7
	*30–45 min*	85.0 ± 5.8	87.2 ± 4.7	88.7 ± 3.6 ^1,4,5,6^	86.3 ± 5.2	87.0 ± 4.4 ^4^
	*45–60 min*	83.0 ± 6.3	84.5 ± 3.5 ^2^	85.4 ± 4.4 ^2,3^	84.4 ± 4.2	84.4 ± 3.7 ^3^
	*60–75 min*	83.9 ± 6.2	84.8 ± 5.2	86.3 ± 4.2 ^3^	84.2 ± 5.0	85.6 ± 3.3
	*75–90 min*	84.3 ± 6.3	85.9 ± 4.9	86.0 ± 5.0 ^2,3^	84.2 ± 5.7	86.0 ± 3.4

HR peak [%HR_max_]	*0–15 min*	93.7 ± 3.3	95.5 ± 2.7	95.2 ± 2.6	95.3 ± 2.7	94.3 ± 3.2
	*15–30 min*	94.2 ± 3.3 ^4^	95.8 ± 2.9	95.5 ± 2.3	96.2 ± 2.6 ^4^	94.6 ± 2.9
	*30–45 min*	94.0 ± 2.9	95.4 ± 2.9	95.9 ± 2.4 ^4^	95.9 ± 2.5 ^4^	95.1 ± 3.3
	*45–60 min*	93.1 ± 3.2 ^2^	93.4 ± 2.7	94.5 ± 2.9 ^3^	93.4 ± 2.9 ^2,3,5^	94.2 ± 3.7
	*60–75 min*	94.1 ± 3.0	94.8 ± 3.3	95.2 ± 2.5	95.7 ± 3.1 ^4^	94.6 ± 3.7
	*75–90 min*	93.9 ± 3.1	95.1 ± 3.3	94.8 ± 2.8	94.7 ± 3.0	94.9 ± 3.1

HRZ3 (70–80%/HR_max_) %/total time	*0–15 min*	16.1 ± 13.4	11.5 ± 10.2	9.4 ± 9.3 ^5^	9.8 ± 8.8	12.5 ± 10.8
	*15–30 min*	16.2 ± 14.1	10.0 ± 10.2 ^5^	9.4 ± 10.0 ^5^	11.2 ± 10.7	11.1 ± 10.4
	*30–45 min*	15.3 ± 15.6	8.2 ± 10.0 ^4,5^	6.2 ± 8.6 ^4,5,6^	11.6 ± 12.2	9.9 ± 10.2
	*45–60 min*	19.4 ± 14.7	16.8 ± 10.3 ^3^	13.5 ± 11.2 ^3^	16.1 ± 12.7	17.1 ± 12.7
	*60–75 min*	17.4 ± 14.1	18.7 ± 13.8 ^2,3^	14.2 ± 11.4 ^1,2,3^	17.1 ± 10.9	13.8 ± 11.2
	*75–90 min*	16.9 ± 14.2	14.9 ± 13.8	12.8 ± 10.9 ^3^	17.5 ± 14.8	12.3 ± 9.2

HRZ4 (80–90%/HR_max_) %/total time	*0–15 min*	50.9 ± 17.1	48.1 ± 17.1	44.7 ± 19.6	47.6 ± 22.0	55.3 ± 16.9
	*15–30 min*	45.3 ± 17.5	45.9 ± 20.9	40.2 ± 18.7	46.0 ± 20.4	54.0 ± 19.7
	*30–45 min*	47.6 ± 19.4	47.8 ± 19.8	39.4 ± 20.1 ^4^	44.4 ± 20.1	52.3 ± 23.6
	*45–60 min*	49.4 ± 17.8	57.0 ± 14.9	47.2 ± 16.2 ^3^	55.8 ± 18.9	57.1 ± 19.3
	*60–75 min*	47.6 ± 16.1	48.3 ± 18.4	42.9 ± 15.6	49.2 ± 17.2	57.2 ± 18.8
	*75–90 min*	47.9 ± 17.6	49.4 ± 19.4	45.2 ± 16.3	48.9 ± 18.0	54.7 ± 17.3

HRZ5 (90–100%/HR_max_) %/total time	*0–15 min*	26.4 ± 21.9	36.1 ± 22.7	41.7 ± 25.7 ^3^	35.5 ± 27.0	27.4 ± 22.0
	*15–30 min*	32.0 ± 25.6	41.5 ± 26.8 ^4^	48.7 ± 25.3 ^4,6^	37.8 ± 27.1	31.8 ± 24.1
	*30–45 min*	31.3 ± 27.0	40.4 ± 24.8 ^4^	52.7 ± 25.4 ^1,4,5,6^	39.3 ± 27.0	35.2 ± 29.0
	*45–60 min*	22.4 ± 21.7	22.5 ± 17.5 ^2,3^	33.8 ± 23.4 ^3^	22.9 ± 22.3	20.7 ± 21.5
	*60–75 min*	27.7 ± 23.1	28.2 ± 24.0	39.4 ± 23.7 ^3^	27.4 ± 23.7	26.2 ± 22.6
	*75–90 min*	28.3 ± 23.3	32.1 ± 26.1	37.2 ± 24.4 ^2,3^	27.5 ± 24.4	29.9 ± 23.0

TD = total distance covered during match, HSR = High-speed running distance covered during match, VHSR = very high-speed running distance covered during match, SPR = sprint distance covered during match, HR = heart rate, HRZ3–5 = heart rate zones 3–5, CD = central defenders, FB = full backs, CM = central midfielders, WM = wide midfielders, FW = forwards.

Statistically significant difference (p < 0.05) compared to 1 = 0–15 min period, 2 = 15–30 min period, 3 = 30–45 min period, 4 = 45–60 min period, 5 = 60–75 min period and 6 = 75–90 min period.

There were some statistically significant differences between 15-min periods in HRmean with FB, CM, and FW, and in HRpeak with CD, CM, and WM. Time spent in HRZ3 was higher during the last three 15-minute periods compared to first three periods in all positions, but statistically significant differences were observed only with FB and CM. Only statistically significant difference in HRZ4 was found in CM who had higher time during period 45–60 minutes compared to period of 30–45 minutes. In general, time spent in HRZ5 was higher in the first three periods than the last three periods, but statistically significant differences between periods were found only in FB and CM.

## DISCUSSION

The aim of the study was to identify positional differences in match demands of national level women’s football and to investigate whether positional match demands change during a match. The main finding was that positional differences identified in match demands were similar to those reported in previously in elite players [1, 11, 12]: total distance was highest in CM and WM and lowest in CD. VHSR and SPR distances and number of accelerations and decelerations were highest in WM and FW and lowest in CD. Between the first and second halves of the matches, statistically significant differences were observed only in CM who showed reduction in TD and HSR distance, and in CD who increased VHSR. However, significant decrement in TD and HSR distance and number of decelerations were observed in comparisons of 0–15 min and 75–90 min in all positions. In addition, position-specific differences between 15 min periods in combined VHSR & SPR, number of decelerations and HR related variables were found. Although significant differences were observed only for some positions and variables, the internal load increased towards the end of the halves while the external load decreased towards the end of the match. Apparently, the present study is in line with the previous studies players experience fatigue towards the end of the match [37].

Average TD per position in matches varied from 9275 to 10797 meters (average 10134 m). These TDs are similar than reported in previous studies from national and elite level players [1, 9–11, 14]. Comparing different positions, CM and WM covered the highest TD, CB the lowest, as in previous studies [1, 8, 9, 11]. While there were no major differences in the current sample of national level players’ TD compared to values reported with elite level players, the distances covered in VHSR and SPR zones were noticeably lower than reported previously from elite players [1]. This finding is supported by other studies which have shown that TD is more stable between different competition levels, whereas the amount of high-intensity running increases in-line with competition level [2, 3, 32]. One explanation for differences in VHSR and SPR distances between levels can be that physical qualities are related to high-intensity running covered during match [33, 36]. Indeed, typically elite players are shown to perform better in general fitness tests than lower-level counterparts [38, 39].

This study found HSR of FB to be significantly lower than that of both WM and FW. This differs from previous studies that have shown the amount of HSR to be similar between FB, WM and FW players [1, 11, 12]. In men, FB high-intensity running demands are shown to increase the most compared to all other positions [40] and most likely similar increase will happen also with national level female players in the future. Consistent with previous studies distance covered in VHSR and SPR zones was highest for WM and FW and lowest for CB and CM [1, 11, 12]. When comparing match demands between positions we do not know if players who run the most are maybe accustomed to running more, or may be selected based on their physical characteristics, for example to meet the higher demands of WM position found in present study. A challenge exists in establishing the causal relationship between playing position and running demands. It is possible that players who run the most may be selected to those positions which have a higher running demand as found in this study.

In contrast to previous studies [7, 26], statistically significant differences in number of accelerations and decelerations between positions were found. WM and FW accumulated the highest total amounts and CD the lowest, whereas previously no differences in number of accelerations and decelerations have been observed between positions [5, 7]. Further comparison with other studies is difficult due to differences in research methods, thresholds, and positional categories. For example, in studies by Ramos et al. [7] and Trewin et al. [8] there is no WM positional categorisation.

In line with previous studies, HR of outfield players in matches averaged 86% of HRmax and statistically significant difference was only observed between CM and CD. Mean HR of 86% corresponds with about 75% of maximal oxygen uptake [15, 17, 27], which indicates that aerobic energy system is heavily taxed in national level match play. Players spent most of their time in HRZ4 (49%) and HRZ5 (33%). Time spend in HRZ5 appears to be a very similar to the elite levels (32% vs. 33% respectively) [31]. The time spent in different HR zones differed slightly from the reports by Krustrup et al. [33] and Panduro et al. [14], in which no differences were observed between positions in the time spent in different HRZ. Compared to elite level players, national level players could produce smaller VHSR and SPR outputs with a similar internal response. One logical explanation could be that the national level players have lower-level physical characteristics compared to elite players. Physical tests were not included for the present study and thus the effect of physical characteristics on performance remains speculative.

The decrease in mean HR from the first to the second half was 1–2%, which corroborates previous observations [3, 30, 31], although there were no significant differences between halves in any other position than CM. When internal load, in terms of HR variables, decreased slightly from the first half to the second, it is logical that a similar decrement was also observed in the external load variables. Statistically the only significant decrements from first to second half was found in CM TD and HSR. On average, TD decreased from first to second half about 2%, which is less than previously reported ~5% [4, 5]. VHSR and SPR distances remained unchanged or even increased from first to second half. The number of decelerations decreased an average of 5% from the first to the second half and the number of accelerations decreased an average of 2%. Still there was much variation and the acceleration rates for CD and WM increased in the second half. Previously VHSR, SPR [9, 26], acceleration and deceleration counts have decreased in the second half of games [5].

External load variables varied between 15-min periods. TD was logically highest at the beginning of the match between 0–15 mins as in previous studies [4, 5], likely because match fatigue had not yet accumulated. Furthermore, TD was generally lower in the last two 15-minute periods similarly to Hewitt et al. [4]. As in previous studies, combined VSHR and SPR was relatively evenly spread between the 15-minute periods in all positions [4, 5], except for WM during the first 15-minute. HSR decreased significantly during a match, and all positions had decrement from 0–15 to 75–90-minute periods as reported in other studies [3, 4, 6] regardless of different thresholds used. In the present study, a general decreasing trend in deceleration counts was observed regardless of the position, but acceleration counts did not decrease significantly, except for CM.

The mean HR is generally increased towards the end of the halves. Within the 15–minute periods, time spent in both the first and second halves in HRZ5 increased towards the end of the halves, which may be related to concurrent increase in HR. The time in HRZ4 was evenly distributed during the match. This demonstrates that the internal load increases towards the end of the halves while the external load seems to decrease. As mentioned before, this could be a sign of players fatigue because of the physical load of the game.

The strengths of the study were that data regarding both external and internal match load were collected and analyzed. As analysis was done in 15-minute periods, the present study can offer more detailed information about possible signs of decrements in female players’ position-specific match demands. Some limitations were also identified in the study. Playing positions were self-reported and no further confirmation (video analysis) was completed by the authors. The study could not consider factors influencing movements of an individual on the pitch, such as teams’ playing style or formations, level of opponent or the score of the match [13]. Furthermore, players motivation, fatigue, recovery time from previous match and conditions (e.g., temperature and weather conditions, surface), effect of away / home game [13, 18] were not controlled. The present study aimed to assess the changes in physical demands during competitive matches and thus, in present study we compared halves and 15-min fixed periods, in line with several previous studies [5, 7]. Demands of fixed periods are not as high as the most demanding passages/ worst case scenarios quantified by using rolling average method [41] and for that reason the findings of present study represent only changes in national level women’s match demands and not absolute peak demands. The most demanding passages/ worst case scenarios are extensively documented in men’s football [42] and in the future similar analysis should be completed with women.

## CONCLUSIONS

In summary, women’s national level football matches require a wide variety of physical demands and the differences between positions are aligned with the elite level. In general, match load can be interpreted as high, although the intensity of the national level matches appears to be lower compared to the international level, especially in terms of very high-speed and sprint running distances. Physical demands in terms of external load appears to decrease during the match, especially after 60 minutes of play, while internal load seems to increase. Findings of present study followed previous studies, which have shown that demands of very high-speed running and sprint running increase in-line with competition level, while total distance remains more consistent.

### Practical applications

From a coaching point of view, it would be important to improve players capability of very high-speed running and sprinting, which would prepare national level players to perform at elite level. Furthermore, position-specific match demands need to be taken into account in individualized training prescription. For example, central midfielders total distance covered during the match is usually very high, so the amount of running in training sessions should also be high. Wide midfielders and forwards cover the highest amount of running meters in very high-speed and sprinting intensities so these players should train more high intense actions. Also, it may be worth assigning players with certain physical profiles to specific positions where their physical abilities align with physical demands.
